# Recreational Athletes’ Use of Performance-Enhancing Substances: Results from the First European Randomized Response Technique Survey

**DOI:** 10.1186/s40798-022-00548-2

**Published:** 2023-01-08

**Authors:** Ask Vest Christiansen, Monika Frenger, Andrea Chirico, Werner Pitsch

**Affiliations:** 1 Department of Public Health, Aarhus University, Aarhus, Denmark; 2 Institute for Sport Science, Saarland University, Saarbruecken, Germany; 3 European Institute for Socioeconomy, Saarbruecken, Germany; 4 Department of Psychology of Development and Socialization Processes, “Sapienza” University of Rome, Rome, Italy

**Keywords:** Doping, Recreational sport, Prevalence, Randomized Response Technique, Europe, Performance enhancement

## Abstract

**Background and Aim:**

Measuring the prevalence of doping in recreational sport is difficult. However, to fit their initiatives, National Anti-Doping Organizations are interested in knowing the numbers, so their scarce resources are not wasted. The present study aimed to estimate the prevalence of doping and over-the-counter medicine use for performance enhancement among recreational athletes in eight European countries.

**Design:**

A survey covering + 200 sports aimed at recreational athletes 15 years and older was distributed via social media to sports clubs and individuals in eight European countries. To overcome social desirability bias, we applied indirect questioning by using the Randomized Response Technique and asked for the use of over-the-counter medicine and doping for the year 2019.

**Results:**

The prevalence of the use of over-the-counter medications for performance enhancement was estimated at 10.4%. We differentiated between the concept of “doping” as the behavior to enhance performance in a certain sport and the concept of “a doper” as a property of a person. The prevalence of dopers in recreational sport was found to be 0.4%, with 3.1% male and 0% female dopers. Responses were separated into four categories: “Artistic sports,” “Combat sports,” “Games,” and “CGS sports” (i.e., sports measured in centimeters, grams, and seconds). The overall prevalence of doping in recreational sports was found to be 1.6%, and the results from Artistic and CGS sports did not differ significantly from this. However, in Games we found an estimated doping prevalence of 6.9%.

**Discussion:**

The estimates for the prevalence of dopers and doping in this study do not equal Anti-Doping Rule Violations as stipulated by the World Anti-Doping Agency. Still, while doping is not absent in recreational sport in Europe, it appears to be a low frequent phenomenon. Also, the differences in doping prevalence between the sports categories might reflect structural and competition-related differences, rather than differences in the logic of the sporting competition or discipline-related subcultures.

**Conclusion:**

While few recreational athletes appear to use illegal drugs to enhance performance, those who do use them are more often men than women. Yet, 1 in 10 recreational athletes uses over-the-counter medication for performance enhancement and more than 4 out of 10 use medication for other reasons than performance enhancement when doing sports. The highest doping prevalence was found in the sub-category of Games, which can likely be attributed to competition-related differences between the categories. Therefore, research on doping in recreational sports needs tailored approaches to come to a better understanding of the phenomenon.

**Supplementary Information:**

The online version contains supplementary material available at 10.1186/s40798-022-00548-2.

## Key Points

1. The prevalence of the use of prohibited performance-enhancing drugs among recreational athletes in Europe appears to be very low.
2. Use of prohibited performance-enhancing drugs is higher among men compared to women and in Games compared to other sports categories.
3. Because of the differences between recreational and elite sport in organizational and competitive structure, a tailored approach is needed to study doping in recreational sport.

## Introduction

Measuring the prevalence of doping is notoriously difficult. Being publicly disapproved, doping is mostly concealed behavior. Therefore, people who do dope will often not respond honestly when asked if they are—even when guaranteed anonymity. In elite sports, there are various ways to estimate doping prevalence, including: (1) testing from blood, urine, or hair, (2) official investigations (governmental, WADA, NADOs, police, other), (3) accounts from for instance athletes and journalists, and (4) surveys using direct or indirect questioning [1, 2]. More recently, wastewater analysis has also been discussed as a potential approach [3, 4]. While estimating doping prevalence in elite populations is challenging, it only becomes more difficult when attending to populations of recreational athletes. For instance, boundaries of the relevant groups of athletes are vague, to approach them is difficult, respondents’ concept of doping is unclear, and no baseline data from for instance anti-doping testing are available. It is thus not surprising that we only know little about recreational athletes’ use of performance-enhancing drugs. To be fair, there are studies that have examined primarily the use of anabolic steroids in gym and fitness environments, as these settings have been assessed to constitute a separate problem regarding drug use in sports [5–7]. While prevalence figures for such groups can be useful for targeted initiatives, they are not of much relevance if the aim is to understand doping in recreational sport more broadly. Knowledge of recreational athletes’ use of performance-enhancing drugs is thus vague and fragmented.

This study aimed to address this knowledge gap, by estimating the prevalence of doping among recreational athletes in Europe.

The present research extends from a previous study, which aimed to “review the existing doping prevention interventions […] which are aimed at sports people, and report on good practices” [8]. Here, the researchers surveyed all EU’s 28-member state’s National Anti-Doping Organizations (NADOs) charged with anti-doping in recreational sports on their assessment of successful interventions. Additionally, a subsample of NADO and sport governing body representatives were interviewed in depth about specific approaches and interventions in their organization or federation. Although more than 85 percent of the NADOs found doping prevention among recreational athletes to be “somewhat or very important when compared to elite-level athletes,” it was also clear that they struggled to identify clear examples of good practice. Thus, a central conclusion was that very little is known about what strategies are effective in preventing doping in recreational sport, as there “is very limited research on the doping problem in competitive recreational sport,” and because “we do not yet have a good understanding of prevalence in various recreational sports” [8].

Prompted hereby, the present study aimed to assess the use of doping among recreational athletes in Europe. More specifically, the aim was to examine the prevalence of doping in recreational sports in eight European countries through indirect questioning by using the Randomized Response Technique (RRT). RRT is a useful technique to investigate sensitive questions where social desirability bias can be expected to distort the reports, and when the sample is expected to be larger than 500–1000 individuals. Thus, if the central question in a survey is “Did you buy cucumber last month,” RRT would not be an advantageous method, whereas it is if the question is “Did you cheat with your tax report last year?”

For this study, sensitive questions were asked for four items: The use of over-the-counter medications for performance enhancement, the use of medication for training or for competition for purposes other than performance enhancement, the use of prohibited substances for performance enhancement, and the use of prohibited substances for image enhancement.

### Terminology

The application of vague concepts can create problems when measuring “doping in recreational sport.” It is therefore necessary to consider the application of the terms “doping,” “recreational sport,” and “recreational athlete.” We opted for a social scientific rather than a legal or theoretical approach to the definition of “doping.” As a result, during the inquiry, it was the respondents’ understanding of doping that was applied, and not the legal definition of the term used by the World Anti-Doping Agency (WADA), or a theoretical definition stipulated by the researchers. We asked explicitly about the use of substances that the respondent believed to be prohibited in their sport. Therefore, if a respondent used a substance which they did not consider to be prohibited it would lead to a “no” answer for the doping question, while the use of a substance, which the respondent thought to be prohibited would lead to a “yes” answer, irrespective of whether the substances in question was in fact prohibited or not. The survey’s social scientific approach thus measures doping in the intentional or moral sense (doping behavior), rather than doping in the legal sense. The prevalence results therefore reflect European recreational athletes’ own understanding of doping and not actual anti-doping rule violations (ADRV) as stipulated by WADA.

We adopted a similar approach with respect to the concepts “recreational athlete” and “recreational sport.” We asked what sports the respondents practiced as a recreational athlete, for how long they had practiced the sport, and at what level they practiced it. Questions concerning the use of medications were related to sport, and not to general health problems. However, we also inquired about the use of substances associated with sports for other purposes than performance enhancement, such as to reduce pain, accelerate recovery, control menstruation, or improve mood. Rather than “performance enhancing,” “performance enabling drugs” may be the appropriate term here. Again, such drugs may be doping in the legal sense of the term, but we explicitly inquired about recreational athletes’ use of medicines that were not intended to enhance performance, and thus could not be considered doping, as stipulated here.

Despite those efforts, there is no way of knowing how respondents understood the key concepts when surveyed. Data arise from their understanding of the concept of “doping,” “prohibited substance,” “recreational athlete” and “recreational sport,” which may only partly overlap with WADA’s definitions as well as with the researchers’ intentional definition. While this should be considered when interpreting the results, it is a challenge that is unavoidable when doing empirical research on this topic. Cautions must therefore be taken when stakeholders, sport policy makers and sport governing bodies assess the results we present.

### Literature Review

For elite sport, the largest systematic review of studies investigating doping prevalence, surveyed the literature from 1975 to 2019 and found 105 relevant studies. The authors assessed the quality of the studies based on 17 criteria and found 20 studies of high quality. Most studies were conducted after 2010 and 10 studies employed the indirect survey techniques like the RRT. In general, studies employing indirect questioning techniques were assessed to have higher quality, than studies using other approaches [2].

**Fig. 1:**
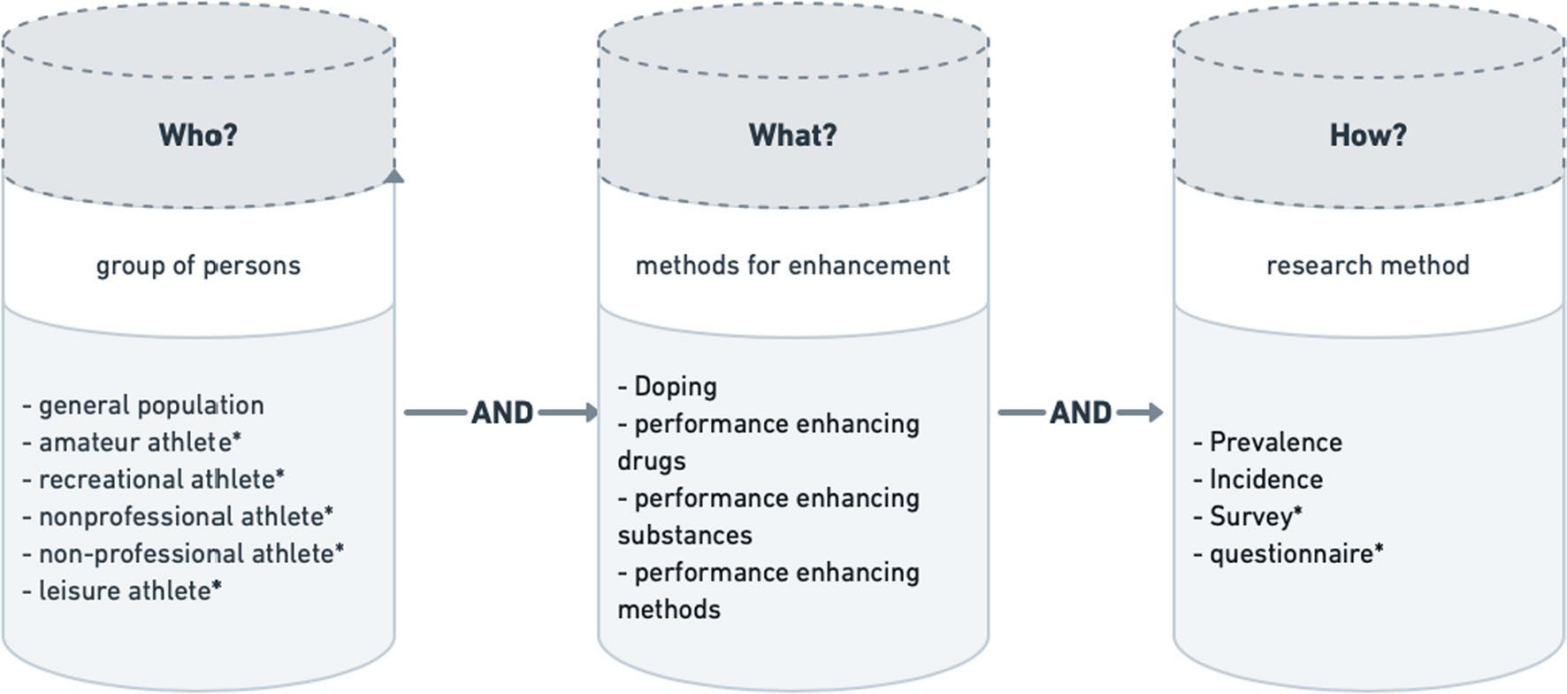
Literature search procedure. Combinations of terms were used for the search process in the literature review. Categories were linked with AND while search terms within the categories were linked with OR. The term combinations are made up of Who? (target group of the study), What? (used substances in a study), and How? (used method of the study). The respective terms of the category are listed in each case as example terms in the figure

For this study on doping in recreational sport, a systematic literature review was conducted to put the present research in context (for search items see Fig. 1, for the search procedure refer to Additional file 1).

The search generated 963 records, that were relevant for further screening procedure. Hereafter duplicates were excluded and so were studies that did not measure prevalence, studies that were not primary, studies that did not concern recreational sport, and studies that did not examine doping or performance-enhancing drugs. After this procedure, 119 articles remained. When filtering this number for studies that did not address an overall recreational athlete population (but rather specific sports or certain subgroups like for instance triathlon, students, or bodybuilders), and for studies that addressed specific (compound classes of) drugs, just three relevant studies were left. However, these either concerned a specific country [9], a limited age scope [10] or focused on methodological comparisons [11]. We therefore believe the present study is the first to survey the use of doping and performance-enhancing drugs in recreational sports in a larger multi-national region.

## Methods and Design

### Randomized Response Technique

**Fig. 2:**
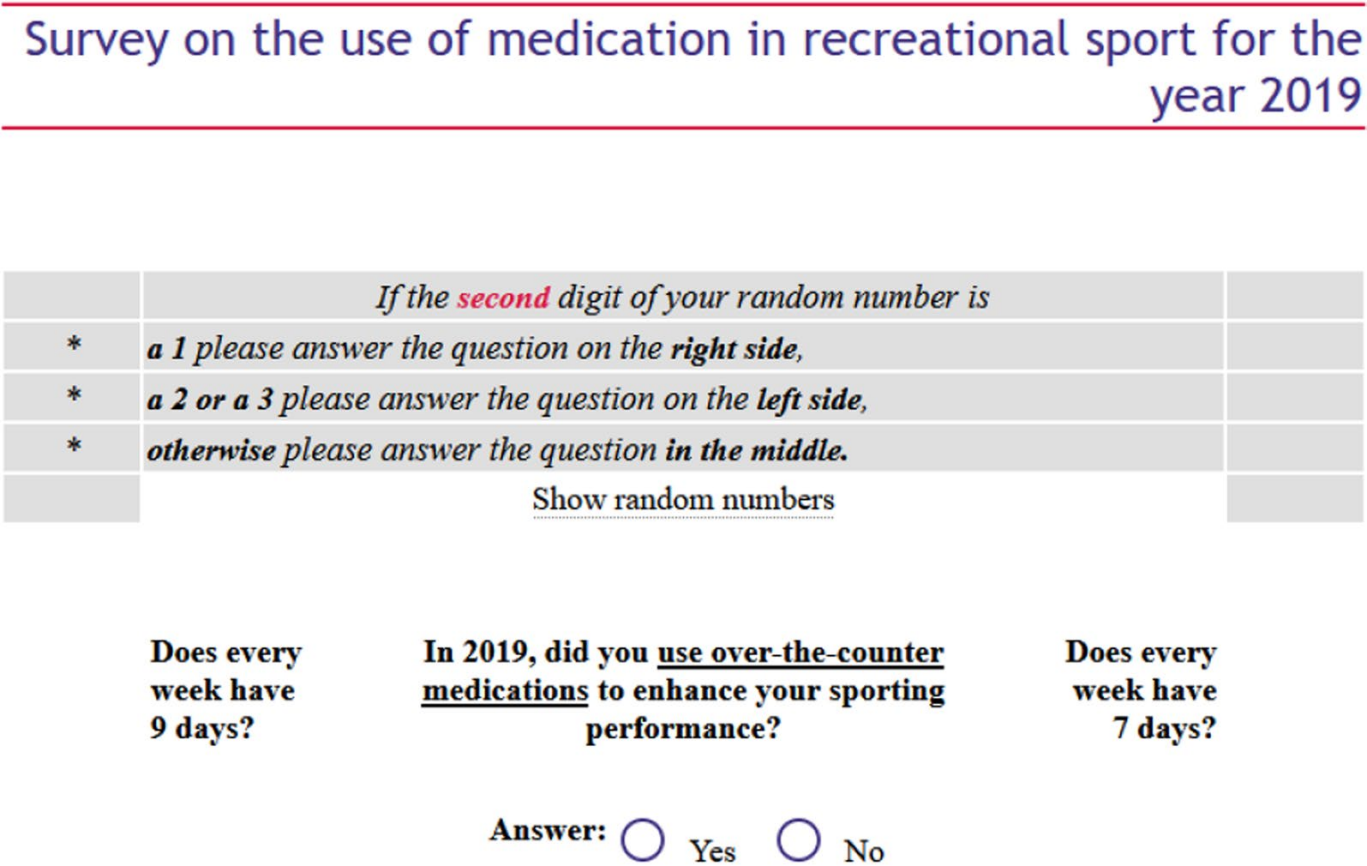
Example of an RRT question from the survey

**Fig. 3:**
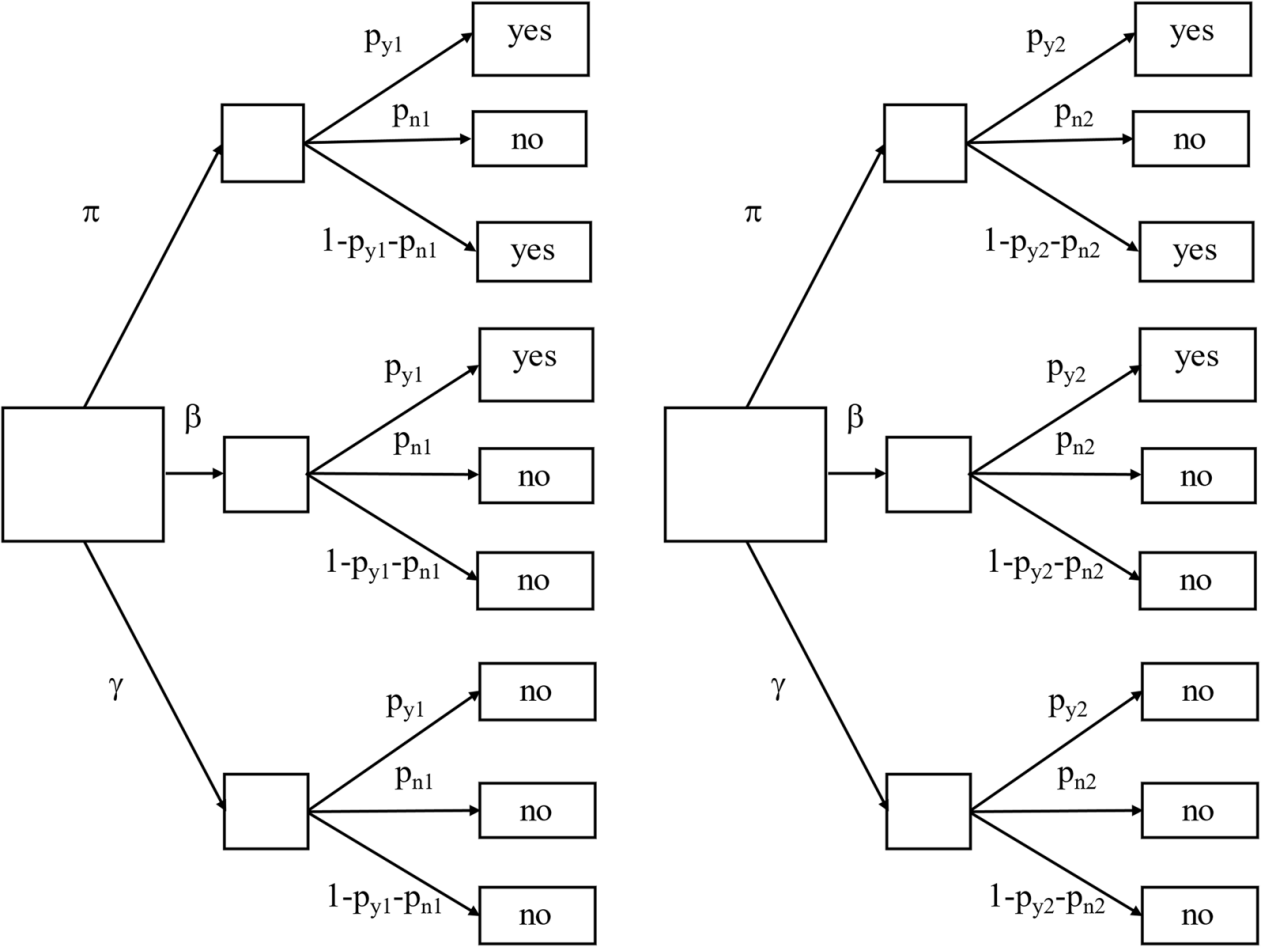
Probability diagram for the RRT with directed INC detection for false “no” answers

**Fig. 4:**
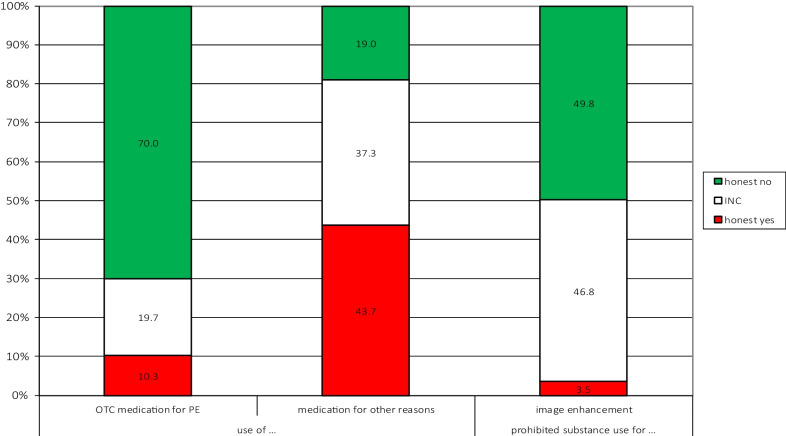
Estimates for the use over-the-counter medications performance enhancement (left), use of medication for training or for competition for purposes other than performance enhancement (center) and for the use prohibited substances or methods to enhance image (right). INC = Instruction Non-Compliance, OTC = Over-The-Counter, PE = Performance Enhancement

**Fig. 5:**
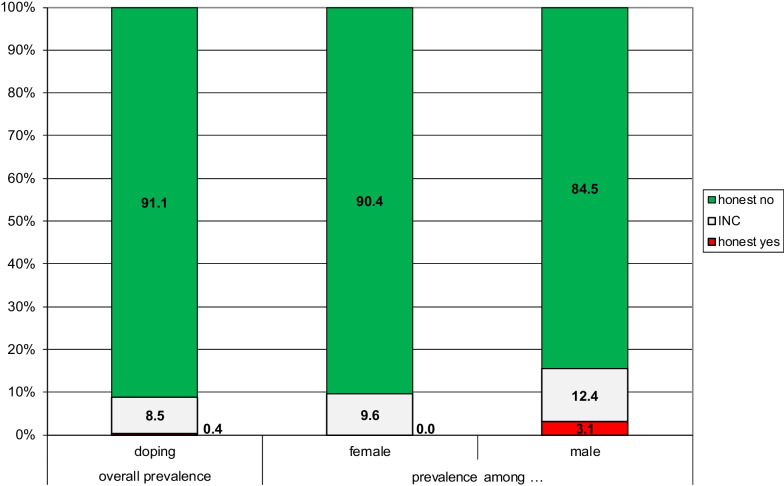
Estimates for the prevalence of dopers in the overall population and among females and males. INC = Instruction non-compliance

**Fig. 6:**
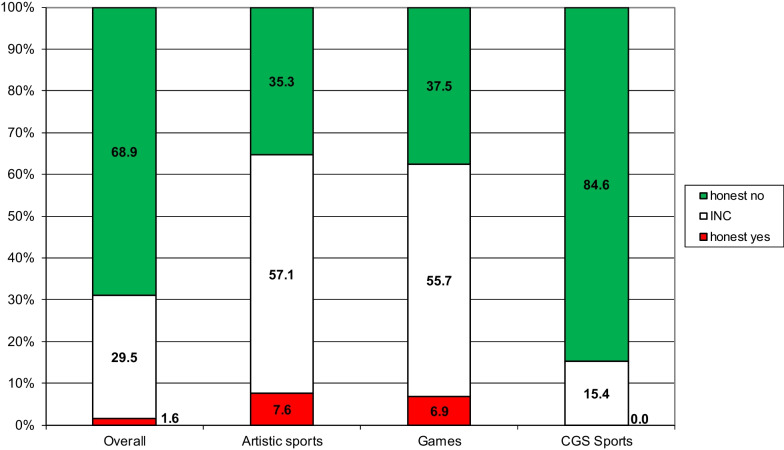
Estimates for the prevalence of doping overall and in Artistic sports, Games, and CGS sports. CGS = sports measured in centimeters, grams, seconds. INC = Instruction Non-Compliance

Because the doping issue is sensitive and admitting to use can be compromising, the survey used the randomized response technique (RRT) for questions on doping and use of medication. The rationale for this was twofold. First, the RRT has been shown to generate more reliable responses than those obtained by direct questioning [2, 12–15]. Second, using RRT ensures comparability of the results with other RRT-based doping surveys in recreational sports [16–18] and in elite sports [2, 19–21]. The primary reason why RRT generates more reliable results is that the method reduces social desirability bias, i.e., the tendency of survey respondents to answer questions in a manner that will be viewed favorably by others [22–25]. In the present study, this would result in underreporting of “bad” or undesirable behavior, such as the use of prohibited medicine for performance enhancement. RRT does not remove social desirability bias altogether, but if the respondent follows the instruction provided, the method gives a perfect assurance against unwanted exposure of their potential undesirable behavior, thereby reducing respondents’ inclination to be influenced by social desirability. The method works by the additional instruction given to respondents when answering a sensitive question. In our case, the respondent was first instructed to select one of five randomly generated 5-digit numbers. The numbers were generated in a way that ensured equal probabilities of the figures 0 to 9 on all 5 digits. This was to prevent sequence effects which could otherwise have led to biased estimates (see below). The respondents were instructed to use the same 5-digit number throughout the survey. Second, the respondent was given the option to either write down the number (or copy-paste it to their laptop) or have the questionnaire software save the list of numbers for them. This was to eliminate suspicions that the researchers could trace the respondent’s choice of random number. Third, the following instruction was given: “If the last digit of your random number is 1 or 2, please answer the question to the right. If the last digit of your random number is 3, 4 or 5, please answer the question to the left. Otherwise, please answer the question in the middle.” The question to the right was: “Does a week have 7 days?” The question to the left was: “Does a week have 9 days?” And the question in the middle was the sensitive question, for instance: “In 2019, did you knowingly use prohibited substances or methods to enhance your sporting performance?” As it appears, depending on the last digit of the random number, respondents will either answer the sensitive question or a corresponding harmless question. A respondent who complies with the instructions will always answer “yes” to the question to the right, and always “no” to the questions to the left.

The process is illustrated in Fig. 2. A respondent who understands the instructions will also realize that if the instructions are followed a certain proportion of respondents will always reply “yes,” whereby an honest “yes” will not attract attention. Since the researchers do not know the random number generated for the respondent, they cannot make any inferences from a “yes” answer regarding the respondent’s actual behavior. A “yes” may be the result of the respondent answering the question to the right (“Does a week have 7 days?”), or the sensitive question in the middle. The researchers will never know.

However, because the researchers know the distribution from which the random number is generated, they can derive the probability that the respondent is instructed to answer the sensitive question. From this, the estimated proportion of people in the population exhibiting the characteristic (here, respondents who intentionally used prohibited substances) can be calculated.

Despite the instructions, some respondents still do not comply with the procedure [12, 26–28]. They are “Instruction-Non-Compliant” (INC). They may deliberately be INC, they may not understand the instruction, or they may simply be making errors. Disregarding the reason, the fact that INC occur reduces the accuracy of the estimate. To control for such biases, the “INC detection model” has been developed [29, 30]. INC detection assumes that RRT estimates of certain specific shares of the population of responders are independent of the probability to answer the harmless questions or the sensitive question. To detect INC, the sample is randomly split into two (normally equally sized) subsamples with different probabilities (Fig. 3). With these two groups, researchers can estimate three population proportions, namely (1) the rate of honest-yes responders, (2) the rate of honest-no responders, and (3) the rate of INC responders.^1^ In this study the probabilities for forced yes and forced no answers were *p*_1y_ = 0.1, *p*_1n_ = 0.2, *p*_2y_ = 0.3, *p*_2n_ = 0.2. These probabilities were selected to maximize the share of honest responders and thereby maximize the statistical efficiency of the estimator for honest-yes and honest-no responses. The cost of this is an increased variance of the INC estimator [30].

The best estimates for the three population shares are calculated by a maximum likelihood estimation. There are, however, cases when the estimate contravenes the mathematically “artificial” marginal conditions, that none of the shares can be above 100% or below zero. For these marginal cases, the remaining estimators and the likelihood of the data are calculated under the condition that one or two of the estimated parameters equal zero. The solution is the one with the highest likelihood. The procedure is explained in detail in the work by Feth et al. [30] (For the R-Code for the present study, refer to Additional file 3). Due to this unnatural limitation of the estimators, the distribution of yes answers is typically heavily skewed by the replacement of negative solutions from the maximum likelihood estimation with 0. For INC and honest no, this replacement of “illegal” solutions with 0 also leads to skewed distributions while there is an additional effect. As “no” answers can only be honest no or INC, the number of “no” answers will in these cases be fully assigned to the parameter which has not been restricted to 0 (these effects can be seen in the distribution of parameter estimations in Additional file 4). Therefore, we use nonparametric bootstrapping to estimate confidence intervals and for hypothesis testing [31].

The RRT method was chosen after discussing the advantages and disadvantages when compared with direct questioning. The reduction in social desirability bias when using RRT has been substantiated in several studies since its introduction in 1965 [for an overview, see@@ [13, 32]. Yet, some scholars have been skeptical about the method, addressing how the intended effect of the RRT depends on (1) the sensitivity of the question under study [33], (2) the respondents’ level of education for understanding the instructions [12, 15], (3) the respondent’s trust in the protection of their anonymity through the RRT [34].

Evidently, indirect questioning methods will add a cognitive load to respondents, which might lead to mistakes when answering [15, 33, 35]. Such mistakes (e. g. misunderstanding the randomization instruction) would lead to random false answers for both yes and no answers. For no answers, this was accounted for by using the no-INC-detection method, which was designed to measure the share of non-compliant no answers no matter if they were deliberate or due to mistakes. For false yes answers, this detection was not possible because of the sample size.

After careful balancing of advantages and disadvantages, we decided to use RRT for questions on doping, image enhancement, and use of medication, as we assessed these issues to be sensitive for recreational athletes. Additionally, other studies on doping and medicine in recreational and elite sport have used similar methods, which makes comparisons easier. Finally, indirect questioning was recommended by the WADA working group on doping prevalence [2, 9, 16–18, 21, 36].

### Survey Questions and Dissemination

The original idea was to measure the point prevalence of doping in recreational sports in Europe in the autumn of 2020. However, as most sports were shut down during the COVID-19 pandemic, we could not ask respondents about their current behavior. After postponing for some months, and still no sign of a forthcoming general European reopening of sports and societies, we decided to run the survey in the spring of 2021 and inquire respondents about their behavior in 2019. Obviously, this entails a risk of recall bias, but due to time limitations of the study period, we had to accept this.

#### Language and Translation

To have a representation of northern, central, and southern Europe in the sample, eight European countries were included in the survey: Norway, Denmark, UK, Germany, Spain, Italy, Greece, and Cyprus. To assist with language issues and troubleshooting, an academic contact person was assigned for each country. The authors covered Denmark, Germany, and Italy, and four European colleagues were invited to cover the remaining five countries (one covering both Greece and Cyprus).

We worked from an English language template where questions, formulations and single words were discussed multiple times to get the best possible phrasing. The survey was then translated from English to six other languages (Greek for Greece and Cyprus, Danish, Norwegian, German, Italian, and Spanish). After translation, the academic partners checked the survey for comprehensibility, compared the version of their language to the English template, and ran small pilots with peers and students.

The questionnaire in the different languages is available at https://fp.socioeconomy.eu/index.php.

#### Survey Dissemination

The survey was disseminated to recreational athletes aged 15 years and older, primarily via snowball sampling using social media platforms. We engaged student assistants to disseminate the survey in each country (the Greek student covering both Greece and Cyprus). Each student assistant had direct contact with their academic partner. The student assistants established a network where they could share dissemination tactics, experiences, problems, and concerns regarding the dissemination. Still, the success of the students in terms of how many survey responses they generated varied greatly (see results below). During the active 12-week survey period, there were weekly meetings with the academic partners to update each other on the progression of the survey.

### Data Quality Control

After data collection, the researchers assessed the data for untrustworthy data that were then deleted. This would, for instance, be records where the respondent reported to be born in 1929 and was still in school or born in 2004 and have obtained a doctorate degree as the highest level of education. Additionally, the time to answer the RRT questions was checked. Had respondents used less than 15 s to answer the first RRT question, the response was deemed untrustworthy. Likewise, data were deleted if the respondent used two seconds or less on the subsequent RRT questions @@[See Ref. [37] for further information on data quality control].

### Weighting Procedures

To calculate the results, we applied weighted statistics. Weights for individuals were calculated to correct for the biased distribution in the number of records per country, by gender, and age in our dataset. Weights were calculated for each question separately to address different levels of question or item nonresponse.

For the data included, weights were selected to approach an overall population of recreational athletes in the eight participating countries as estimated from Eurostat population descriptions and the most recent Eurobarometer survey on sports and physical activity and for Norway from the Norwegian national statistical bureau [38–40] [for details of the weighting procedure,@@ see [37].

## Results

In total, 17,324 clicks on the link to the survey were registered. There were 8,146 records with data, of which 7260 were from respondents reporting to be recreational athletes. However, as athletes were asked for more than one sport that was assessed independently, 9562 records, covering 218 sports, were obtained. After data quality control, the final number of records to be analyzed was 9365. As respondents were asked about the use of prohibited substances in up to two sports, 6167 records addressing doping behavior were obtained.

The results presented cover only the subsample of recreational athletes who indicated that they had played at least one sport in 2019. As respondents do not always give complete reports, numbers do not necessarily add up to the same figures in all tables.

### Sample Structure by Age, Gender, and Country

Almost twice as many males as females participated in the survey. In addition, there were more records from younger age-groups, under 35 years. Older age-groups (above 65 years) and other genders (intersex, non-binary, and transgender)^2^ were only marginally present in the dataset (Tables 1, 2).

**Table 1:** Distribution of respondents by age, gender, and country

*By gender*																
Female			Male		Non-binary				Transgender			Intersex				Final number for analysis
1867			3822		(14)				(11)			(3)				5689
*Age class*																
15–24	25–34			35–44		45–54	55–64			65–74				75–84		
2129	1226			994		809	405			(129)				(19)		5536
*Country*																
DK		UK		ESP		GER		GR/CY			IT		NO		Other	
2521		227		1102		428		334			560		351		(225)	5523

DK = Denmark, UK = United Kingdom, ESP = Spain, GER = Germany, GR = Greece, CY = Cyprus, IT = Italy, NO = Norway

**Table 2:** Distribution of respondents by country

Gender	Country								
	DK	UK	ESP	GER	GR/CY	IT	NO	Other	Total
Female	651	84	365	233	157	213	81	83	1867
Male	1846	138	717	187	187	338	264	145	3822
Total	2497	222	1082	420	344	551	345	228	5689

DK = Denmark, UK = United Kingdom, ESP = Spain, GER = Germany, GR = Greece, CY = Cyprus, IT = Italy, NO = Norway

Respondents were asked to list up to four sports, which they played in 2019. A list of 155 different sports were shared for the eight countries, while an additional 17 sports were considered relevant only in some countries. Respondents reported to be playing 149 of these sports. However, as the survey had an option to add a sport not listed, 78 respondents added 59 additional sports, raising the number of sports played by respondents to 208. The most frequently chosen sports were (in descending order) jogging/running, cycling, swimming, fitness, and football (soccer). Table 3 shows the distribution of respondents by the number of sports played.

**Table 3:** Distribution of respondents by the number of sports they played in 2019

Number of sports played	1	2	3	4
Respondents	3,404	1,478	561	323

Respondents were asked to indicate the level of competition in 2019, for the sports they registered, and for how long they had played the sport. The level of competition was categorized as follows:

1. I didn’t compete in 2019
2. Local level
3. Regional level
4. National level
5. International level.

To avoid survey fatigue, RRT questions were limited to two sports, even if a respondent reported to play more than that. This was done by prioritizing their sports. First by the level of competition, with the higher priority for the highest competitive level (first order) and by how long the respondent had played the sport (second order). Thus, RRT questions for doping in 2019 were asked for the two sports with the highest priorities.^3^

### RRT Questions

The questionnaire contained four or five RRT questions, depending on whether the respondent played one or more sports. Below the questions are ordered as they appeared to the respondents in the questionnaire:

1. In 2019, did you use over-the-counter medications to enhance your sporting performance?
2. In 2019, did you use medication for training or for competition for purposes other than performance enhancement (e.g., for pain relief, injury recovery, or to control sleep, mood, or menstrual cycle)?
3. When participating in [*the sport*] in 2019, did you knowingly use prohibited substances or methods to enhance your sporting performance?
4. In 2019, did you knowingly use prohibited substances or methods to enhance your image?

As mentioned, if the respondent played more than one sport in 2019, the third question would be asked twice, one time for each of the two sports given the highest priority.

For questions 1, 2, and 4, the resulting best estimates are shown in Fig. 4 (complete result tables including 95% confidence intervals are provided in Additional file 2).

The first two questions addressed behavior that is not prohibited, but that may nevertheless be socially disapproved (Fig. 4, the two bars to the left). Approximately 10 percent of the population indicate that they have used over-the-counter medication for performance enhancement. However, as instruction non-compliance is 20 percent, the true estimate for the prevalence of over-the-counter medication for performance enhancement is at least 10 percent but could be up to a maximum of 30 percent. As regards the use of medication for training or competition for purposes other than performance enhancement, 44 percent report such use. Noteworthy, instruction non-compliance for this question is 37 percent, so while 44 percent is the lower limit of the true prevalence, it could possibly be up to 81 percent.

While these questions addressed legal behavior that might be viewed as unfavorable, the last two questions addressed behaviors that are prohibited in sport. In the last question, respondents were asked if they knowingly had been using prohibited substances or methods to enhance their image (Fig. 4, bar to the right).

Expectedly, the best estimate for honest-yes answers is lower than for the first two questions, namely around 3 percent. However, the estimate for instruction non-compliance of 47 percent is extraordinarily high for a RRT question. The fact that the question addressed prohibited behavior most likely contribute to the high INC. But the very concept the question addresses seem insufficiently clear, which has likely raised INC. In hindsight, we realize that for large proportions of the responding population the question was not straightforwardly understandable. Supporting this assumption was several questions from respondents inquiring about the meaning of the wording “prohibited substances or methods to enhance your image.” In fitness and gym environments, and especially in the bodybuilding community, the concept of performance enhancement overlaps with the concept of image enhancement, and it therefore makes sense to most individuals in this sport. However, the idea of “image enhancing drugs” is incongruous to most recreational athletes like cyclists and football players and thus makes little sense to them. Consequently, because of the incoming inquiries about the meaning of the term “image enhancement” in association with the unusual high rate of INC we decided to omit this variable from further analyses.

### RRT Questions on Prohibited Performance-Enhancing Drugs

When interpreting our results, it is important to differentiate between the concept of “doping” as a behavior to enhance one’s performance in a certain sport and the concept of “a doper” as a property of a person. Thus, the prevalence of dopers in a population of recreational athletes should not be confused with the prevalence of doping for a given sport. To give an example: A person reports to be cycling and doing gymnastics. The person will then be given the RRT question on the use of prohibited performance-enhancing substances for both sports. Let us imagine the person reports the use of prohibited performance-enhancing substances for cycling, but not for gymnastics. Ergo, the respondent gave the answer “yes” to doping in cycling and “no” to doping in gymnastics. This matters for the prevalence results. Because the person reported to be using prohibited performance-enhancing substances s/he would contribute to the estimated percentage of “dopers” when reporting on the overall prevalence of doping among recreational athletes. However, when reporting on sports (or categories of sport) the person would only contribute to the prevalence of doping in cycling (and the category of sports cycling belongs to) and not in gymnastics (or the category of sports gymnastics belongs to). To approach the concept of a doper, we used only one record per individual. For respondents with more than one sport and one answer being “yes” and one being “no,” we selected the record with the “yes” answer.^4^ If both answers were the same, we selected the record randomly. As mentioned, the questions on the doping topic concerned the intentional use of prohibited performance-enhancing substances or methods. In Fig. 5, left bar, the overall prevalence of dopers in the eight participating countries is shown.

As can be seen, the best estimate for the prevalence of dopers doing recreational sports in Europe is close to zero. There are less than a half-percent “honest-yes” responders and a relatively low rate of instruction non-compliance of 8 percent. Differentiating between females and males nuances the picture slightly. Whereas doping is negligible among females (with 10 percent INC), 3 percent of males report to be doping (with 12 percent INC). Phrased conservatively, we can be confident that 90 percent of female and 85 percent of male recreational athletes in the eight participating countries do not intentionally use prohibited substances to enhance their sporting performances.

### Analysis by Sports Category

Since the number of records generated was insufficient to estimate doping prevalence for individual sports, we needed to cluster sports into relevant categories. To guide this procedure, we followed the vulnerability thesis for doping suggested by Loland [41]. The assertion is that: “For any athletic performance goes that […] the higher significance of basic bio-motor qualities and the lesser significance of technical and tactical skills, the more vulnerable a performance becomes to doping” [41]. Following this logic, we categorized the 208 sports into four categories (and a residual category), based on the structure and aim of the sport, and on how performance in competition is determined. The four categories are: “Artistic sports” (e.g., dance and gymnastics), “Combat sports” (e.g., judo, karate, boxing), “Games” (e.g., football, tennis, volleyball), “CGS sports” (sports measured in centimeters, grams, and seconds, e.g., athletics, cycling, swimming) and the residual category, “Other.” The categories are useful as it is relatively easy for researchers to determine where each sport belongs. Also, the categorization has been applied in previous research on doping and prevalence [20, 21, 42]. Finally, such categorization is sensible since potent drugs indeed affect bio-motor qualities to a much larger extent than technical and tactical skills.

The raw, weighted, and relative distribution of responses by sports category is given in Table 4.

**Table 4:** Raw, weighted, and relative distribution of responses by sports category

	Artistic	Combat	Games	CGS	Other
Records	1197	234	2431	5106	361
Weighted by gender, age, and country	1505	191	1928	5376	569
Relative weighted distribution (%)	15.7	2.0	20.1	56.2	5.9

CGS = sports measured in centimeters, grams, seconds

Almost 60 percent of responders practiced a sport that fell into the CGS category, 20 percent did Games, and 15 percent Artistic sports. This corresponds to the finding that jogging/running, cycling, football, and fitness were found to be among the most popular sports.

While looking at sports, in general, is interesting from an overall perspective, many would like to know the situation in their sport category. Because of too low response rates, prevalence estimates could not be calculated for combat sports and the residual category. As mentioned, the number of records for sports exceeds the number of respondents because some respondents practiced more than one sport and therefore replied twice to the doping question. Accordingly, the overall prevalence for doping presented in Fig. 6 (left bar) differs from the overall rate of dopers presented in Fig. 5 (left bar).

For Artistic and CGS sports, the “honest yes” estimation did not differ significantly from the overall category (Fig. 6). Only for Games, a significant difference (at a significance level of 5%) was found. This indicates that recreational athletes who played Games in 2019 were more prone to intentionally use prohibited performance-enhancing substances compared to recreational athletes from other sports categories (see Additional file 2 for details on the calculations).

However, while we did find statistically significant differences in the “honest no” estimates between the sports categories, this was likely a consequence of the differences in the INC estimate. Thus, differences in the “honest no” estimate could not, with certainty, be attributed to differences in doping behavior in the different sports categories. This was only the case for the difference in the “honest yes” estimate for Games.

## Discussion

This study shows that doping among European recreational athletes is what Thomas Sandøy refers to as a “low frequent phenomenon” [43]. While the overall prevalence of dopers was less than half a percent, we could not measure any doping among females and found a 3 percent prevalence of male dopers. Although this is not the same as doping being absent, the idea that doping has contaminated sports at all levels is a myth. European recreational athletes do not in large proportions intentionally use prohibited drugs to enhance their sporting performance.

From the perspective of the NADOs, who regard doping prevention among recreational athletes to be somewhat or very important when compared to elite-level athletes [8, 44], the findings could be interpreted as evidence that they have succeeded in conveying the message that recreational athletes should not dope. On the other hand, critics, of what has been labeled mission creep among anti-doping organizations [45, 46], could equally well interpret the results as confirmation that doping is indeed not a problem in recreational sport, and that the anti-doping organizations should therefore focus on the elite and leave recreational athletes to themselves. Since there is a lack of evaluations of the impact of NADO’s interventions and anti-doping campaigns for recreational athletes, the former interpretation is unfounded [8]. The latter interpretation rests on criticism toward undue interference in people’s private affairs. Based on our findings, such criticism is warranted as regards the implementation of extensive and expensive anti-doping test regimes targeting recreational athletes (which are indeed within the jurisdiction of 60% of European NADOs [8]), whereas the employment of relatively inexpensive educational campaigns with far-reaching potential is less problematic [47].

The findings also suggest that there are reasons to distinguish between men and women on this issue. We know that men are more oriented toward competition, that they are more focused on social dominance and hierarchies than are women [48–50]. The use of doping is a means to perform better in competitions (formal as well as informal) that very concretely measures performances, ranks athletes, and thus establishes social hierarchies. Therefore, to the extent that doping is being used in recreational sport, it is no surprise that it is used by men, not women. However, we also know that historically, women, compared to men, have had restricted access to sport, and the findings here may be a residual effect of that [51]. Additionally, the found a higher prevalence of male dopers could also be methodological and caused by a combination of different variables and their interactions (see Limitations section).

As mentioned, the doping prevalence results reported here do not equal Anti-Doping Rule Violation (ADRV) as defined by WADA [52]. We asked if respondents had intentionally used prohibited substances to enhance their performance, and thus addressed only one of 10 items classified as ADRVs. Additionally, most respondents do not know the content of the WADA-list and therefore we do not know to which extent recreational athletes’ understanding of “prohibited substances” overlaps with WADA’s legal concept of doping [11, 52]. Still, the results show that most recreational athletes do not intentionally use substances they think are prohibited.

At first sight, the finding of a higher prevalence of doping in Games compared to Artistic and CGS sports is surprising. In the context of elite sports, it is generally acknowledged that CGS sports are more vulnerable to doping than Games [41]. While there is a direct relationship between doping and performance in for instance cycling, this is not the case in for instance football, which is also reflected in WADA’s analysis of ADRVs [53]. Yet, a drawback of the applied categorization is that it does not consider athletes’ approach to their sport, but only the sport’s structure. In recreational sport, this may be more significant for doping behavior than how vulnerable to doping the sport, or the sport’s category, is. However, the structural difference between Games and other sports when practiced recreationally may explain the finding. While Artistic and CGS sports can be practiced with other objectives than competition (to preserve or improve health or to socialize), Games typically take place as competitions, disregarding the primary motivator and level of practice. Subsequently, compared with the other sports categories, formal structures and a higher degree of organization is needed for Games in recreational sport. In line with this, only 18% of respondents playing Games indicated that they did not compete in 2019, while this was the case for 47% in the CGS category and 75% in the Artistic. Therefore, the found differences in doping prevalence between the sports categories might well reflect structural and competition-related differences, rather than differences in the logic of the sporting competition or discipline-related subcultures.

Consequently, the finding of a higher doping prevalence in Games compared to CGS or Artistic sports should not be taken to indicate that doping is prevalent in amateur football but absent in cycling or fitness. While the categories reflect groupings of sports with similar structure and ways to measure performance, the result for the category cannot be generalized to all sports within the category. Competitive recreational cyclists and gym enthusiasts could very well be doping, even if it cannot be measured on the level of their sports category.

The structural and competitive differences across the categories have consequences for the study of doping in recreational sport. Looking closer at the data, three important aspects appear: (A) The Artistic sports category is dominated by respondents doing fitness. This is not the case in elite sports, where fitness is largely absent from the category of artistic sports. (B) The Games category is dominated by respondents competing and where formal structures to measure performances are in place. This mirrors elite sport. But whereas in elite sport Games are considered less vulnerable to doping than are CGS sports, here Games is the category of competition which is subsequently reflected in a higher doping prevalence. (C) The CGS category, usually considered to be vulnerable to doping, has many records from respondents who do not compete and thus do not formally measure performance, resulting in a lower doping prevalence. Taken together these findings expose two things: First, the four sports categories have different content and meaning in recreational sports than they have in elite sport. Second, the vulnerability thesis [41] has little explanatory power in recreational sport. Consequently, doping emerges in other ways in recreational sports than in elite sport, and therefore the phenomenon deserves its own research approaches.

Assessing our results in light of the few relevant questionnaire-based prevalence studies found in the literature review reveals how difficult it is to make comparisons between studies with different designs. We found 0.4% to be dopers in a population where many practiced sports without competing—recall that 47% and 75% did not compete in the CGS and Artistic categories, respectively. Compared to this, Frenger and colleagues found 4% dopers, when they asked for doping among respondents who had competed during the last season [9]. Lentillon-Kaestner and Ohl found a 2.7% lifetime doping prevalence in a sample population consisting of French Swiss school athletes [11]. Finally, Özdemir and colleagues [10] found a doping prevalence of 8.0% in a sample population comprised mainly of young males where half had an athletic license and with wrestling, weightlifting, boxing, and running being the dominant sports [10].

Even if the present study only found few dopers, approximately 10 percent report to be using over-the-counter medications for performance enhancement, and the use of medications for other reasons than performance enhancement is close to 45 percent. This indicates that the idea of being opposed to doping is not the same as being opposed to the use of medicine when practicing sport. When 10 percent of the respondents use over-the-counter medications for performance enhancement, it illustrates that the competitive element of sport is also important for recreational athletes. They too want to win or perform well when they do sport. Accordingly, not everyone doing recreational sport has health or the social benefits of sport as their prime motivator. However, when 44% of European recreational athletes use medicine for other reasons than performance enhancement, it is a powerful illustration of how important it is to be physically active. The respondents want to play their sport even if their back is aching or their knee hurts. Obviously, a negative interpretation of this could be that recreational athletes have become dependent on medicine to live their lives. A positive interpretation, however, would conclude that the finding illustrates how important physical activity is to the European citizens. They want to do sport and be active even when faced with the adversaries of an aging or aching body. And luckily, legal remedies exist that can help them being active.

## Limitations

There are several limitations to the study. While we believe this is the first survey on the use of doping and performance-enhancing drugs in recreational sports in a larger multi-national region in Europe, we did not include any countries to represent Eastern Europe. Additionally, the sampling via social media systematically excluded those who do not use social media; participation or attrition rate may also be explained by cultural differences or subcultural readiness to participate in surveys [54]. This is indicated by the uneven distribution of respondents from the eight countries, in that large countries like the UK and Germany had, respectively, 10- and 6-times fewer respondents than a smaller country like Denmark.^5^ Even if the applied weighting procedure corrected for the bias in return rates, it only does so for known biases, such as country, age, and sex. Other things, such as the population distribution by sports or sports categories, the level of athletic success, the rate of club or individually organized sports, or the number of sports played by recreational athletes, are unknown. We do not know if the sample is biased in one or more of these dimensions.

The survey applied the RRT method for all questions concerning performance enhancement and medicine. While we remain confident that the method is superior when it comes to the doping question (see “Methods and Design” section), it is likely that it does not provide better estimates for questions on the use of over-the-counter medication for performance enhancement and for the question of sport-induced use of medication for other purposes than performance enhancement. For these two questions, social desirability likely influences replies to a much lesser degree than is the case for the more sensitive doping question. Therefore, direct questioning might have produced reliable results while additionally it could have reduced the cognitive load on respondents, reduced the large INC, and allowed for more sophisticated statistical analyses. Yet, the rationale for applying the RRT, also for these questions, was to provide data that could be compared to the doping questions and at the same time allow respondents to become familiar with the RRT method before asking the sensitive doping questions. Also, even if it is legal, using medication for performance enhancement might be socially disapproved and the extent to which this affects social desirability bias has, to our knowledge, not been tested empirically.

The study revealed the influences of different variables. For example, we found a higher prevalence in Games than in other sports categories, and we found a higher prevalence of male than female dopers. However, we do not know to what extent these variables are mutually dependent and if there are interaction effects. It is possible that gender is not the influencing variable, but rather the type of sport and the level of competition. Men participate more often in competitions and may play different sports than women.

## Conclusion

This study used the indirect questioning technique, RRT, to assess the prevalence of sport-induced medicine use and the use of performance-enhancing substance among European recreational athletes in 2019. Ten percent of respondents reported the use of over-the-counter medications for performance enhancement, whereas almost 45% indicated to use medicine for other reasons than performance enhancement when playing sports. We distinguished between “doping,” as the use of prohibited substances in a given sport, and “dopers,” as designating individuals intentionally using prohibited substances. While we found an overall prevalence of 0.4% dopers, we saw 3.1% male and zero percent female dopers. Looking at sports rather than individuals, showed an overall doping prevalence of 1.6%. Of the four sports categories, Games was the only one with a higher prevalence than the overall category. Additionally, the differences between recreational and elite sports in organizational and competitive structure signify that the applied sports categories have different content, meaning, and relevance in recreational sports compared to elite sports. Consequently, the vulnerability thesis has less explanatory power in recreational sports than it has in elite sports. Therefore, to come to a better understanding of the phenomenon, doping in recreational sports needs tailored research approaches.

## Supplementary Information

**Additional file 1.** Adapted PRISMA flowchart.

**Additional file 2.** Complete tables, confidence intervals, and results from statistical tests.

**Additional file 3.** R-Code for RRT estimation with “no”-INC-detection.

**Additional file 4.** Skewed distributions of RRT estimators from bootstrap replications.

## Data Availability

Data as well as information on handling of data, calculations, and weighting procedures are available at https://doi.org/10.17605/OSF.IO/JXZA5 [37]. Complete result tables including 95% confidence intervals and R-Code for the estimation of RRT-measured prevalence rates with “no”-INC-detection are provided in Additional file 2 and Additional file 3. Additional information can be requested by inquiry to Dr. Werner Pitsch, we.pitsch@mx.uni-saarland.de.

## Abbreviations

RRT: Randomized Response Technique
NADO: National Anti-Doping Agency
WADA: World Anti-Doping Agency
INC: Instruction Non-Compliance
CGS sports: Sports measured in centimeters, grams, seconds, e.g., cycling, running, swimming
ADRV: Anti-doping rule violation

## Footnotes

1 In the literature, this third proportion is often referred to as “cheaters,” which is unfortunate as it may suggest that they cheated in sport by using prohibited substances or that they deliberately did not follow the RRT instructions. Therefore, we use the term “Instruction non-compliant” or INC responder. Further analysis of the RRT method with INC detection is available in [27, 29, 30].

2 There was a correlation between respondents registering other genders than men or women, and iffy data like “born in 1926 and still in school,” “born in 2004 and having a PhD,” as well as response times below 15 s for the first RRT question.

3 When choosing the highest level of competition to prioritize sports, we accommodate the widely accepted hypothesis that doping is used to give a competitive edge. Thus, the likelihood for someone to use performance-enhancing substances increases with the level of competition. Still, in line with the survey’s aim to address recreational sport, very few respondents reported to be at the international level. And those who did, most often played sports with low participation rates and low public interest in their country. It could for instance be a Dane choosing American football. A sport played by few in Denmark, which would result in players often taking part in international tournaments to compete. Even if they would thus be “international level,” they would still be regarded as amateurs. For this reason, we have not excluded “international level” records from the analysis.

4 The reason for selecting the yes-answer when there was one “yes” and one “no,” is that it has the highest probability of originating from someone who doped. Recall that only some respondents would answer yes because they doped, while (most) others would do so because of the instruction. We made the decision to choose the “yes-answer” because it would guarantee that we did not accidentally discard any responses from dopers and thereby inadvertently would end up with an erroneously low estimate of dopers.

5 The differences in the number of respondents from the eight countries, could of course also be due to differences in strategy, effort, or luck in survey dissemination from the student assistants assigned with this task.

## Code availability

See Additional file 3.
