# Supplementary material for: Recreational Athletes’ Use of Performance-Enhancing Substances: Results from the First European Randomized Response Technique Survey
Source: Sports Med Open. 2023 Jan 8;9:1. doi: 10.1186/s40798-022-00548-2 (PMC9825800; doi:10.1186/s40798-022-00548-2)
Supplement: Supplementary file 1 — Additional file 1. Adapted PRISMA flowchart. [file 40798_2022_548_MOESM1_ESM.docx]

Recreational athletes’ use of performance enhancing substances: Results from the first European Randomized Response Technique survey. *Sports Medicine – Open*, Ask Vest Christiansen: Aarhus University, Monika Frenger, Saarland University, Andrea Chirico, "Sapienza" University, Werner Pitsch: Saarland University, E-mail: [avc@ph.au.dk](mailto:avc@ph.au.dk)

Searched Databases were SportDiscus, PubMed (NLM, PMC Central, Medline), Proquest, Scopus, Web of Science, BISp Surf, Livivio. The PRISMA statement was followed but had to be adapted due to 1) the diversity of studies which did not allow to conduct a Meta analysis and 2) the inclusion of cross sectional and longitudinal studies which had to be handled differently (Moher, Liberati, Tetzlaff, & Altman, 2009).

Records after duplicates removed
(n = 963)

Studies included in further analysis
(n = 123)

Studies included in cross sectional data extraction
(n = 119)

Identified series of publications on longitudinal data
(n = 4)

Additionally identified longitudinal studies
(n = 7)

Full-text articles assessed for eligibility
(n = 224)

Full-text articles excluded, with reasons:
(no prevalence study, n = 23
no primary st., n = 1
no rec.sport, n = 62
no doping, n = 13)

Records screened
(n = 947)

Records excluded
(n = 723)

Records identified through database searching
(n = 708)

Additional records identified through other sources
(n = 257)

Studies/reports included in longitudinal data extraction
(n = 49)

Moher, D., Liberati, A., Tetzlaff, J., & Altman, D. G. (2009). Preferred reporting items for systematic reviews and meta-analyses: the PRISMA statement. *Annals of Internal Medicine, 151*(4), 264-269, W264. doi:10.7326/0003-4819-151-4-200908180-00135
