# Supplementary material for: Recreational Athletes’ Use of Performance-Enhancing Substances: Results from the First European Randomized Response Technique Survey
Source: Sports Med Open. 2023 Jan 8;9:1. doi: 10.1186/s40798-022-00548-2 (PMC9825800; doi:10.1186/s40798-022-00548-2)
Supplement: Supplementary file 2 — Additional file 2. Complete tables, confidence intervals, and results from statistical tests. [file 40798_2022_548_MOESM2_ESM.docx]

Recreational athletes’ use of performance enhancing substances: Results from the first European Randomized Response Technique survey. *Sports Medicine – Open*, Ask Vest Christiansen: Aarhus University, Monika Frenger, Saarland University, Andrea Chirico, "Sapienza" University, Werner Pitsch: Saarland University, E-mail: [avc@ph.au.dk](mailto:avc@ph.au.dk)

# Complete results and significance tests

Low/bca and high/bca are the lower and upper limits of the 95% confidence intervals of the estimators. Confidence intervals were were retrieved from a bootstrapping procedure (2000 boostrap replications) including bcα correction. INC is the estimation of instruction non-compliance.

## Complete results including 95% confidence intervals for data in figure 3

*Table 1: Prevalence estimates and confidence intervals for over-the-counter medications use for performance enhancement and for medication use for purposes other than performance enhancement. Results shown in percentages.*

|  | In 2019, did you use over-the-counter medications to enhance your sporting performance? | | | In 2019, did you use medication for training or for competition for purposes other than performance enhancement? | | |
| --- | --- | --- | --- | --- | --- | --- |
|  | estim. | low/bca | high/bca | estim. | low/bca | high/bca |
| honest yes | 10.3 | 4.3 | 17.1 | 43.7 | 35.2 | 51.4 |
| INC | 19.7 | 7.9 | 30.8 | 37.3 | 25.7 | 50.0 |
| honest no | 70.0 | 56.6 | 85.5 | 19.0 | 1.7 | 33.8 |

*Table 2: Prevalence estimates and confidence intervals for using prohibited substances or methods for image enhancement. Results shown in percentages.*

|  | In 2019, did you knowingly use prohibited substances or methods to enhance your image? | | |
| --- | --- | --- | --- |
|  | estim. | low/bca | high/bca |
| honest yes | 3.5 | 0.0 | 9.5 |
| INC | 46.8 | 38.0 | 55.6 |
| honest no | 49.8 | 38.2 | 60.3 |

## Complete results including 95% confidence intervals data for data in figure 4

Table 3: *Prevalence of dopers in recreational sport in percentages and confidence intervals. Results shown in percentages.*

|  | When participating in [your sport] in 2019, did you knowingly use prohibited substances or methods to enhance your sporting performance? | | |
| --- | --- | --- | --- |
|  | estim. | low/bca | high/bca |
| honest yes | 0.4 | 0.0 | 5.9 |
| INC | 8.5 | 0.0 | 16.9 |
| honest no | 91.1 | 82.3 | 100.0 |

Table 4: *Prevalence of dopers among females and males in percentages and confidence intervals. Results shown in percentages.*

|  | Female | | | Male | | |
| --- | --- | --- | --- | --- | --- | --- |
|  | estim. | low/bca | high/bca | estim. | low/bca | high/bca |
| Honest yes | 0.0 | --- | --- | 3.1 | 0.0 | 10.6 |
| INC | 9.6 | 0.0 | 21.6 | 12.4 | 0.0 | 26.7 |
| Honest no | 90.4 | 78.4 | 100.0 | 84.5 | 66.1 | 97.5 |

## Complete results including 95% confidence intervals and significance tests for data for figure 5

*Table 5: Doping prevalence in sport categories. Results shown in percentages.*

|  | weighted estimates | | | |
| --- | --- | --- | --- | --- |
|  | estim. | low/bca | high/bca |  |
| Overall: honest yes | 1.6 | 0.0 | 5.3 |  |
| Overall: Inc | 29.5 | 22.1 | 36.5 |  |
| Overall: honest no | 68.9 | 60.5 | 77.6 |  |
| Artistic: honest yes | 7.6 | 0.0 | 17.2 |  |
| Artistic: Inc | 57.1 | 42.8 | 71.6 |  |
| Artistic: honest no | 35.3 | 16.1 | 53.4 |  |
| Games: honest yes | 6.9 | 0.4 | 16.1 |  |
| Games: Inc | 55.7 | 42.9 | 67.3 |  |
| Games: honest no | 37.5 | 20.6 | 52.1 |  |
| CGS: honest yes | 0.0 | - | - |  |
| CGS: Inc | 15.4 | 5.8 | 23.0 |  |
| CGS: honest no | 84.6 | 76.5 | 94.7 |  |

*Table 6: Estimates as well as one-sided 95% confidence intervals for differences between sport categories. For positive differences, a positive lower limit of the confidence interval indicates a significant difference. For negative differences, a negative upper limit of the confidence interval indicates a significant difference.*

|  | estim. | low/bca | high/bca |
| --- | --- | --- | --- |
| Comparison cgs sport vs. Other sports | | | |
| Honest yes | -5.21 |  | 0.00 |
| INC | -37.18 |  | -23.98 |
| Honest no | 42.39 | 27.19 |  |
| Comparison games vs. Other sports | | | |
| Honest yes | 6.86 | 0.05 |  |
| INC | 33.69 | 20.91 |  |
| Honest no | -40.54 |  | -26.10 |
| Comparison artistic sports vs. other sports | | | |
| Honest yes | 6.88 | -1.07 |  |
| INC | 31.34 | 16.57 |  |
| Honest no | -38.22 |  | -20.46 |
