# Supplementary material for: Recreational Athletes’ Use of Performance-Enhancing Substances: Results from the First European Randomized Response Technique Survey
Source: Sports Med Open. 2023 Jan 8;9:1. doi: 10.1186/s40798-022-00548-2 (PMC9825800; doi:10.1186/s40798-022-00548-2)
Supplement: Supplementary file 3 — Additional file 3. R-Code for RRT estimation with “no”-INC-detection. [file 40798_2022_548_MOESM3_ESM.docx]

Recreational athletes’ use of performance enhancing substances: Results from the first European Randomized Response Technique survey. *Sports Medicine – Open*, Ask Vest Christiansen: Aarhus University, Monika Frenger, Saarland University, Andrea Chirico, "Sapienza" University, Werner Pitsch: Saarland University, E-mail: [avc@ph.au.dk](mailto:avc@ph.au.dk)

# R-Code for RRT estimation with “no”-INC-detection

Required package: pracma

###############################################################################

# Helper functions

###############################################################################

## Parametervector

# checks the given parameter vectors

# input: all requested parameter vectors but 1

# output: the parameter vectors, complains when sum(parametervectors)!=1

# and when 1 or more parametervectors are not between 0 and 1.

ParameterVector <- function(a, b = NULL, c= NULL, d = NULL, ignoreConstraints = FALSE){

if(nargs() == 1){

if(length(a) == 4){

pv <- a

} else('Input vector has false length')

} else if(nargs() == 3){

pv <- c(a, b, c, (1-a-b-c))

} else if(nargs() == 4| nargs() == 5){

pv <- c(a, b, c, d)

} else('ungueltige Argumente')

if(nargs() !=0 && !(nargs() == 5 && ignoreConstraints == TRUE)){

checkParameterBounds(pv)

checkParameterSum(pv)

}

return(pv)

}

# checkParameterBounds: Returns FALSE if one of the parameters lies outside [0, 1]

checkParameterBounds <- function(pv.v, quiet = TRUE){

names = c('a', 'b', 'c', 'd')

for(i in 1:4){

if((pv.v[i] <0 && abs(pv.v[i]) > .Machine$double.eps) | pv.v[i] > 1){

if(!(quiet == TRUE)){

print(paste('Parameter', names[i], need to lie within [0,1]; tried',

names[i], '=', round(pv.v[i], 3), sep = " "))

}

return(FALSE)

}

}

return(TRUE)

}

# checkParameterSum Returns FALSE if the sum of parameters > 1

checkParameterSum <- function(pv.v, quiet = TRUE){

s = sum(pv.v)

if(abs(s-1) > .Machine$double.eps){

if(!(quiet == TRUE)){

print(paste('Sum of parameter =', s, sep = " "))

}

return(FALSE)

}else{

return(TRUE)

}

}

## likelihood

# Calculates the log-likelihood for re-calculated rates of yes-answers v

# per group given the empirical results kv and Nv

# - v: rate of Yes-Answers per group for a given parameter set

# - kv: empirical Number of 'Yes'-answers per group

# - Nv: number of valid answers per group

likelihood <- function(v, kv, Nv){

return(t(as.matrix(kv)) %*% log(v) + t(as.matrix(Nv - kv)) %*% log(1-v))

}

## RRTAnswers

# Administers the answers from a RRT-questionnnaire with n Groups and N sample size

# Input: dataframe of interest, P1 and P0

# Output: obj

RRTanswers <- function(matrix_, P1_, P0_, answercolumn = 2,

groupcolumn = 1, weightcolumn = 0, bootcharcolumn = 3,

yesvalue = 1, novalue = -1, missingvalue = 0){

if(groupcolumn > ncol(matrix_)){

return('Illegal value for groupcolumn')

}

if(answercolumn > ncol(matrix_)){

return('Illegal value for answercolumn')

}

if(weightcolumn > ncol(matrix_)){

return('Illegal value for weightcolumn')

}

if(bootcharcolumn > ncol(matrix_)){

return('Ungueltiger Wert fuer bootcharcolumn')

}

obj <- list(

"matrix" = matrix_,

"P1" = P1_,

"P0" = P0_,

"answercolumn" = answercolumn,

"groupcolumn" = groupcolumn,

"weightcolumn" = weightcolumn,

"bootcharcolumn" = bootcharcolumn,

"yesvalue" = yesvalue,

"novalue" = novalue,

"missingvalue" = missingvalue,

"numgroups" = length(P1_))

class(obj) <- "RRTAnswers"

return(obj)

}

## Count

# Counts the answers given

# Input: dataframe as returned by RRTAnswers

# Output: kv and NV

# kv: (nx1) vector with the number of 'Yes'answers in the respective RRTgroups

# Nv: (nx1) vector with the number of valid answers in the respective RRTgroups

count <- function(obj, index){

if(nargs() == 1){

index <- seq(TRUE, nrow(obj$matrix))

}

require(tibble)

kv <- vector(mode = "numeric", length = obj$numgroups)

Nv <- vector(mode = "numeric", length = obj$numgroups)

if(obj$weightcolumn == 0){

for(i in 1:obj$numgroups){

kv[i] <- sum(obj$matrix[index,obj$answercolumn] == obj$yesvalue &

obj$matrix[index,obj$groupcolumn] == i)

Nv[i] <- sum(obj$matrix[index,obj$answercolumn] != obj$missingvalue &

obj$matrix[index,obj$groupcolumn] == i)

}

}else{

for(i in 1:obj$numgroups){

kv[i] <- sum((obj$matrix[index,obj$answercolumn] == obj$yesvalue &

obj$matrix[index,obj$groupcolumn] == i) *

obj$matrix[index, obj$weightcolumn])

Nv[i] <- sum((obj$matrix[index,obj$answercolumn] != obj$missingvalue &

obj$matrix[index,obj$groupcolumn] == i) *

obj$matrix[index, obj$weightcolumn])

}

}

return(tibble(kv,Nv))

}

###############################################################################

# Core function for RRT estimation including "no"-INC estimation

###############################################################################

## Estimator for NCD

# input: rrtAnswers-Object and index (index is the rows that are considered for analysis)

# output: calculated parameter vector

estimateNCD <- function(obj, index = 0, ignoreconstraints = FALSE){

if(!isa(obj, "RRTAnswers")){

return("Wrong type of first argument. Should be an RRTAnswers object")

}

if(obj$numgroups < 2){

return("at least 2 subgroups are required")

}

# setting unset subgroups to "all"

if(length(index) == 1){

index <- seq(TRUE, nrow(obj$matrix))

}

require(tibble)

require(pracma)

cnt <- count(obj, index)

lambda <- as.matrix(cnt[1]/cnt[2])

M <- matrix(c((1-obj$P0), obj$P1), 2, 2)

theta <- pracma::mldivide(M, lambda)

# Dataframe P takes the values for alpha to delta in columns a to d

# as well as for the cornercases the logLikelihood in column L .

# Row names refelct the cornercases.

P <- data.frame(matrix(c(rep(0, 35)), nrow= 7, ncol = 5,

dimnames = list(c("CC_NONE", "CC_A", "CC_B", "CC_C",

"CC_AB", "CC_AC", "CC_BC"),

c("a", "b", "c", "d", "L"))))

P["CC_NONE", c(1:4)] <- ParameterVector(theta[1],

1-sum(theta),

theta[2], 0, ignoreconstraints)

if((ignoreconstraints == FALSE) &&

!(checkParameterBounds(P["CC_NONE", c(1:4)]) &

checkParameterSum(P["CC_NONE", ]))){

# CornerCase treatment, 6 CC's

# alpha = 0

M <- obj$P1

c <- pracma::mldivide(M, lambda)

P["CC_A", c(1:4)] <- ParameterVector(0, 1-c, c, 0, TRUE)

# beta = 0

M <- 1-obj$P0-obj$P1

a <- pracma::mldivide(M, (lambda-obj$P1))

P["CC_B", c(1:4)] <- ParameterVector(a, 0, 1-a, 0, TRUE)

# gamma = 0

M = 1-obj$P0

a = pracma::mldivide(M, lambda)

P["CC_C", c(1:4)] <- ParameterVector(a, 1-a, 0, 0, TRUE)

# alpha = beta = 0

P["CC_AB", c(1:4)] <- ParameterVector(0, 0, 1, 0, TRUE)

# alpha = gamma = 0

P["CC_AC", c(1:4)] <- ParameterVector(0, 1, 0, 0, TRUE)

# beta = gamma = 0

P["CC_BC", c(1:4)] <- ParameterVector(1, 0, 0, 0, TRUE)

MBase = matrix(c(1-obj$P0, rep(0,obj$numgroups), obj$P1, rep(0,obj$numgroups)),

nrow = 2, ncol = 4, byrow = FALSE)

# LogLikelihood

for(i in 2:7){

if(!(checkParameterBounds(P[i, c(1:4)]))){

P[i, "L"] <- -Inf

}else{

v <- MBase %*% t(as.matrix(P[i, c(1:4)]))

P[i, "L"] <- likelihood(v, cnt[1], cnt[2])

}

}

this_one <- which(P[c(2:7), "L"] == max(P[(2:7), "L"])) + 1

return(as.vector(P[this_one, c(1:4)]))

}else{

return(as.vector(P["CC_NONE", c(1:4)]))

}

}
