# Supplementary material for: Recreational Athletes’ Use of Performance-Enhancing Substances: Results from the First European Randomized Response Technique Survey
Source: Sports Med Open. 2023 Jan 8;9:1. doi: 10.1186/s40798-022-00548-2 (PMC9825800; doi:10.1186/s40798-022-00548-2)
Supplement: Supplementary file 4 — Additional file 4. Skewed distributions of RRT estimators from bootstrap replications. [file 40798_2022_548_MOESM4_ESM.docx]

Recreational athletes’ use of performance enhancing substances: Results from the first European Randomized Response Technique survey. *Sports Medicine – Open*, Ask Vest Christiansen: Aarhus University, Monika Frenger, Saarland University, Andrea Chirico, "Sapienza" University, Werner Pitsch: Saarland University, E-mail: [avc@ph.au.dk](mailto:avc@ph.au.dk)

# Skewed distributions of RRT estimators from bootstrap-replications


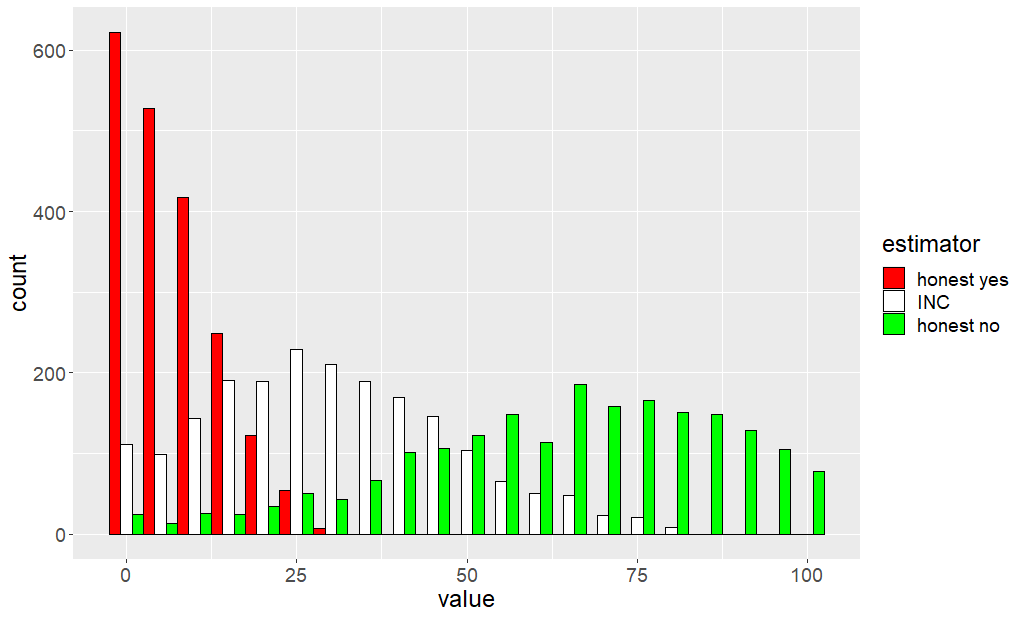


***Fig. S4*** *Distributions of RRT estimators from 2000 bootstrap-replications for an assumed true score for honest yes close to zero and as a result of assigning no-answers to INC in cases of “illegal” solutions for honest no and vice versa.*
